# From Bench to Bedside: Attempt to Evaluate Repositioning of Drugs in the Treatment of Metastatic Small Cell Lung Cancer (SCLC)

**DOI:** 10.1371/journal.pone.0144797

**Published:** 2016-01-06

**Authors:** Zoltan Lohinai, Peter Dome, Zsuzsa Szilagyi, Gyula Ostoros, Judit Moldvay, Balazs Hegedus, Balazs Dome, Glen J. Weiss

**Affiliations:** 1 National Koranyi Institute of Pulmonology, Budapest, Hungary; 2 Department of Clinical and Theoretical Mental Health, Kútvölgyi Clinical Center, Semmelweis University, Budapest, Hungary; 3 National Institute of Psychiatry and Addictions, Budapest, Hungary; 4 Translational Thoracic Oncology Laboratory, Division of Thoracic Surgery, Comprehensive Cancer Center, Medical University of Vienna, Vienna, Austria; 5 Molecular Oncology Research Group, Hungarian Academy of Sciences-Semmelweis University, Budapest, Hungary; 6 Department of Biomedical Imaging and Image-guided Therapy, Medical University of Vienna, Vienna, Austria; 7 Western Regional Medical Center, Cancer Treatment Centers of America, Goodyear, Arizona, United States of America; Univesity of Texas Southwestern Medical Center at Dallas, UNITED STATES

## Abstract

**Backgrounds:**

Based on in vitro data and results of a recent drug repositioning study, some medications approved by the FDA for the treatment of various non-malignant disorders were demonstrated to have anti-SCLC activity in preclinical models. The aim of our study is to confirm whether use of these medications is associated with survival benefit.

**Methods:**

Consecutive patients with pathologically confirmed, stage 4 SCLC were analyzed in this retrospective study. Patients that were prescribed statins, aspirin, clomipramine (tricyclic antidepressant; TCA), selective serotonin reuptake inhibitors (SSRIs), doxazosin or prazosin (α1-adrenergic receptor antagonists; ADRA1) were identified.

**Results:**

There were a total of 876 patients. Aspirin, statins, SSRIs, ADRA1, and TCA were administered in 138, 72, 20, 28, and 5 cases, respectively. A statistically significant increase in median OS was observed only in statin-treated patients when compared to those not receiving any of the aforementioned medications (OS, 8.4 vs. 6.1 months, respectively; p = 0.002). The administration of SSRIs, aspirin, and ADRA1 did not result in a statistically significant OS benefit (median OS, 8.5, 6.8, and 6.0 months, respectively). The multivariate Cox model showed that, besides age and ECOG PS, radiotherapy was an independent survival predictor (Hazard Ratio, 2.151; 95% confidence interval, 1.828–2.525; p <0.001).

**Conclusions:**

Results of drug repositioning studies using only preclinical data or small numbers of patients should be treated with caution before application in the clinic. Our data demonstrated that radiotherapy appears to be an independent survival predictor in stage 4 SCLC, therefore confirming the results of other prospective and retrospective studies.

## Introduction

Lung cancer is the most frequently diagnosed malignancy worldwide and is a leading cause of cancer mortality [1]. Importantly, Hungarians have the world’s highest death rates from lung cancer [2].

Small cell lung cancer (SCLC) is a very aggressive neuroendocrine subtype, and accounts for 15% [1] of all lung cancers. While the number of new agents and treatment options has markedly increased in other cancers, for SCLC, chemotherapy remains the main component of care and no new class of systemic therapy has entered clinical practice in the past three decades [3]. Patients often present with advanced stage at diagnosis. Surgical resection for a patient with advanced SCLC is rarely prospectively planned and serves little clinical benefit [4]. Thus, there is a limited amount of tumor tissue available for molecular analysis and translational research. Therefore, there remains a large, unmet need of new strategies for drug development.

Drug repositioning which is the identification of old drugs for use in a new indication has recently led to more rapid and less expensive drug development due to their known dose and toxicity profile [5]. Based on in vitro and in vivo results of a recent systematic drug repositioning bioinformatics studies, some medications approved by the Food and Drug Administration (FDA) for the treatment of various non-malignant disorders were demonstrated to have anti-SCLC activity in preclinical models [6]. Drug dose levels that displayed anti-cancer activity were similar to those used in the clinic. The side-effect profile of two of these agents, clomipramine and doxazosin, fair better to most chemotherapy for SCLC. Clomipramine, a tricyclic antidepressant (TCA), has pleiotropic effects, such as serotonin and norepinephrine reuptake inhibition as well as antagonism of some G-protein coupled receptors (GPCRs), e.g. muscarinic acethylcholine, histamine H1 and adrenergic α1 receptors) [7]. Doxazosin, a selective α1-adrenergic receptor (ADRA1) antagonist, led to decreased cell survival and inhibition of downstream signaling [6].

The antidepressant fluoxetine, a selective serotonin reuptake inhibitor (SSRI), has demonstrated Ca^2+^ independent apoptosis in cancer cells [8] and acts at the serotonin pathway similarly to the TCAs, disrupting autocrine survival signals involving neurotransmitters and their GPCRs [6, 9].

Statins, commonly used cholesterol-lowering agents in clinical practice, act on the Ras pathway [10], have anti-proliferative, pro-apoptotic, and anti-metastatic effects in SCLC [11]. Statins have been reported to reduce the incidence of lung cancer and also increase the survival of patients with lung cancer [12]. Anti-inflammatory and anti-platelet drugs like aspirin may play an important role in preventing cancer risk and progression possibly by the involvement of cyclooxygenase-2 in the pathogenesis of lung cancer [13].

Nevertheless, the clinical relevance of these drugs for the treatment of metastatic SCLC remains unclear. The aim of our study is to evaluate whether the use of these medications is associated with survival benefit in a large, well-defined cohort of metastatic SCLC patients from a single institution.

## Materials and Methods

### Ethics Statement

The study was conducted based on the ethical standards prescribed by the Helsinki Declaration of the World Medical Association and with the approval of the national level ethics committee (Hungarian Scientific and Research Ethics Committee of the Medical Research Council [ETT TUKEB]). The approval number is 52614-4/2013/EKU. Patient consent is not required and was not obtained for this study, concerning retrospective study of existing data. Patients were de-identified and the clinical information were collected. Patients cannot be identified either directly or indirectly.

### Study Population

Consecutive patients with cytologically or histologically confirmed, metastatic (TanyNanyM1) SCLC evaluated at the National Koranyi Institute of Pulmonology between 2000–2013 were analyzed in this retrospective study. Patients that were prescribed statins, aspirin, clomipramine, SSRIs, doxazosin and prazosin were identified. Information collected included prescription of the above medications, clinicopathological characteristics (including gender, age, Eastern Cooperative Oncology Group performance status [ECOG PS]), applied chemo- and radiotherapy, and overall survival (OS). TNM stage according to the Union for International Cancer Control (7th edition)[14], ECOG PS, and age were evaluated at the time of diagnosis.

### Treatment

Patients were treated first-line with a platinum-etoposide doublet regimen or with a combination of cyclophosphamide, epirubicin, and vincristine (CEV). Radiation therapy (RT) including thoracic RT, prophylactic cranial irradiation (PCI), or whole brain radiation therapy (WBRT) were administered to selected patients. The study and all treatments were conducted in accordance with contemporary NCCN guidelines.

### Statistical Methods

OS was estimated from the time of diagnosis in patients presenting with metastatic stage IV disease, until death or last available follow-up. Date of last follow-up included in this analysis was February 15, 2015. Patients were grouped according to prescribed medications and compared to those in the control group (patients not on any of the highlighted medications). Kaplan-Meier curves and two-sided log-rank tests were used for univariate survival analyses. Age (<70 yrs vs. ≥70 yrs) was considered as a categorical variable. The Cox proportional hazards model was used for uni- and multivariate survival analyses to calculate the hazard ratios (HR) and corresponding 95% confidence intervals (CI). To address the problem of multiple comparisons, Bonferroni’s correction was applied. Thus, with eight confounding variables (e.g. gender, age, ECOG PS, RT, aspirin, statins, SSRIs, and ADRA1 [doxazosin and prazosin were grouped together since both are ADRA 1 antagonists]), p-values less than 0.00625 were considered to indicate statistical significance. All p-values were two-sided. All variables with p-values less than 0.00625 were included in the multivariate analysis. For multivariate survival analyses, the Cox regression model was adjusted for age as a categorical variable (<70 yrs vs. ≥70 yrs), ECOG PS (0–1 vs. >1), statin treatment, and RT. Metric data are shown as median or mean and corresponding range or, in case of OS, as median and corresponding 95% CI. Clinical characteristics of patients prescribed statins compared to the control group or RT vs. non-RT were analyzed by the Chi-square test, while ages were compared using the Student’s t-test. All statistical analyses were performed using the PASW Statistics 18.0 package (SPSS Inc., Chicago, IL, USA).

## Results

The patient clinical characteristics are shown in Table 1 and S1 Table. There were a total of 876 Caucasian patients (508 men and 368 women) with a median age of 61 years (range, 33–86). First-line platinum-etoposide chemotherapy and second-line chemotherapy was administered in 65% and 39.9% of the cases; respectively. The median follow-up was 6.5 months (range, 0–150 months) with 868 deaths out of 876 patients. Aspirin, statins, SSRIs, ADRA1, and TCA were prescribed in 138, 72, 20, 28, and 5 cases; respectively (S1 Table). Statin and aspirin were concurrently prescribed in 36 cases (among them SSRIs and ADRA1 were also administered in 2 and 4 cases; respectively), while aspirin and SSRIs or ADRA1 were concurrently prescribed in 1 and 5 cases, respectively. Gender, age, ECOG PS, and different treatments were tested for predicting OS.

**Table 1:** Major clinical characteristics of metastatic SCLC patients received radiation therapy (RT) versus patients not treated with RT (n = 876).

	RT	Non-RT	p-value
**Total**	299	577	
**Age (mean±SD)**	59.7 ± 8.6	62.5 ± 9	**<0.001**
**Gender**			
** Male**	173 (58%)	335 (58%)	n.s
** Female**	126 (42%)	242 (42%)	
**ECOG PS**			
** 0–1**	218 (73%)	362 (63%)	**0.003**
** >1**	81 (27%)	215 (37%)	
**Chemotherapy**			
** Platinum-etoposide**	224 (75%)	295 (52%)	**<0.001**
** CEV**	73 (24%)	211 (36%)	
** Unknown data/ best supportive care**	2 (1%)	71 (12%)	-
**Radiation therapy**			
** PCI**	30	-	-
** WBRT**	174	-	-
** Thoracic RT**	158	-	-
**Median OS (95%CI) months**	10.0 (8.9–10.9)	4.8 (4.3–5.4)	-

Data shown in parentheses are column percentages. ***ECOG PS***: Eastern Cooperative Oncology Group performance status; ***OS***: overall survival; ***CEV***: cyclophosphamide, epirubicin, vincristine; ***PCI***: prophylactic cranial irradiation; ***WBRT***: whole brain radiation therapy.

**Fig 1:**
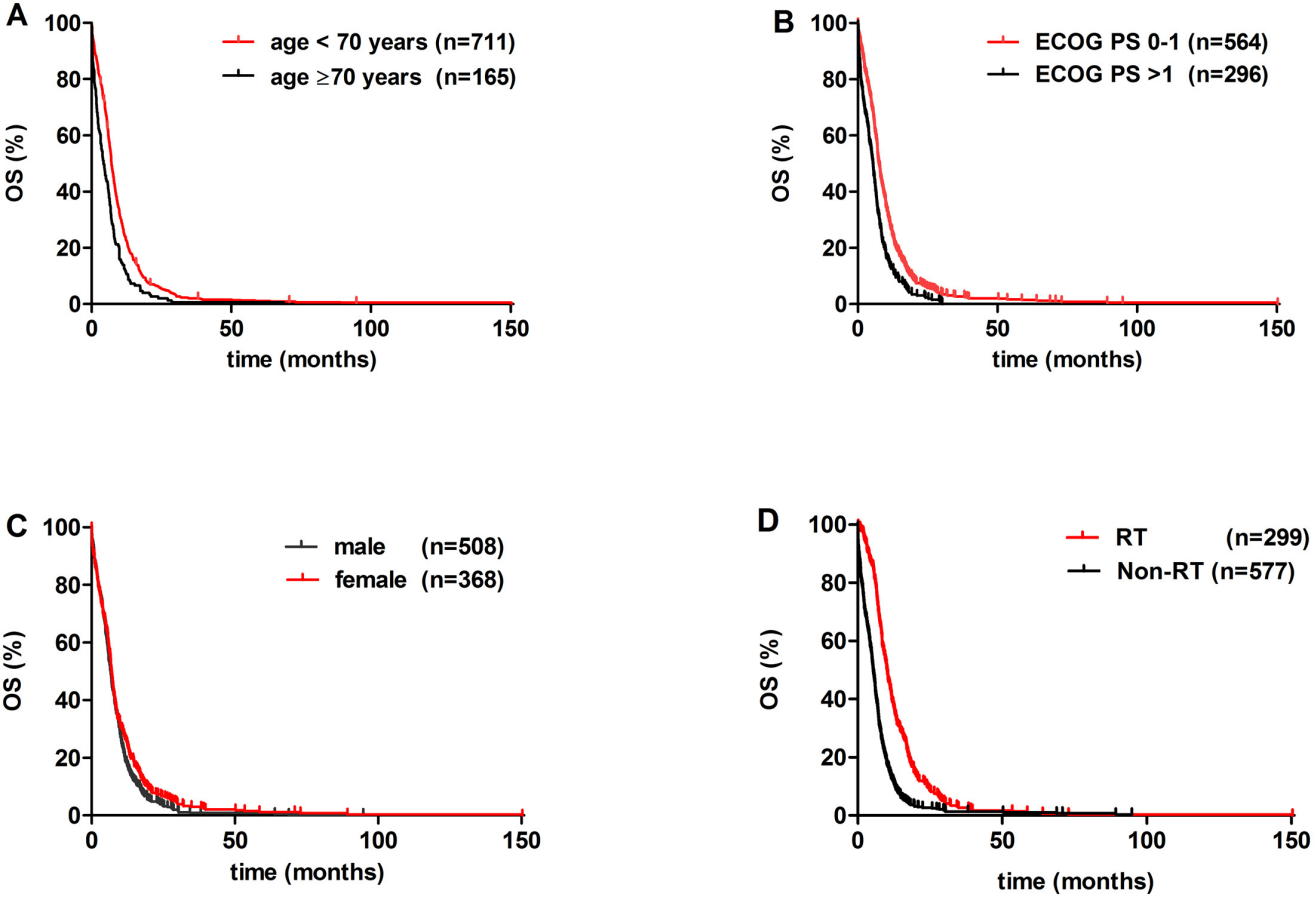
Kaplan-Meier curves for the OS of clinical variables of metastatic SCLC patients. (A) age <70 yrs vs. ≥70yrs (p = 0.001), (B) ECOG PS 0–1 vs. ECOG PS >1 (p<0.001), (C) male (vs. female; p = 0.021), and (D) radiation therapy (RT) vs. patients not treated with RT (p<0.001).

**Fig 2:**
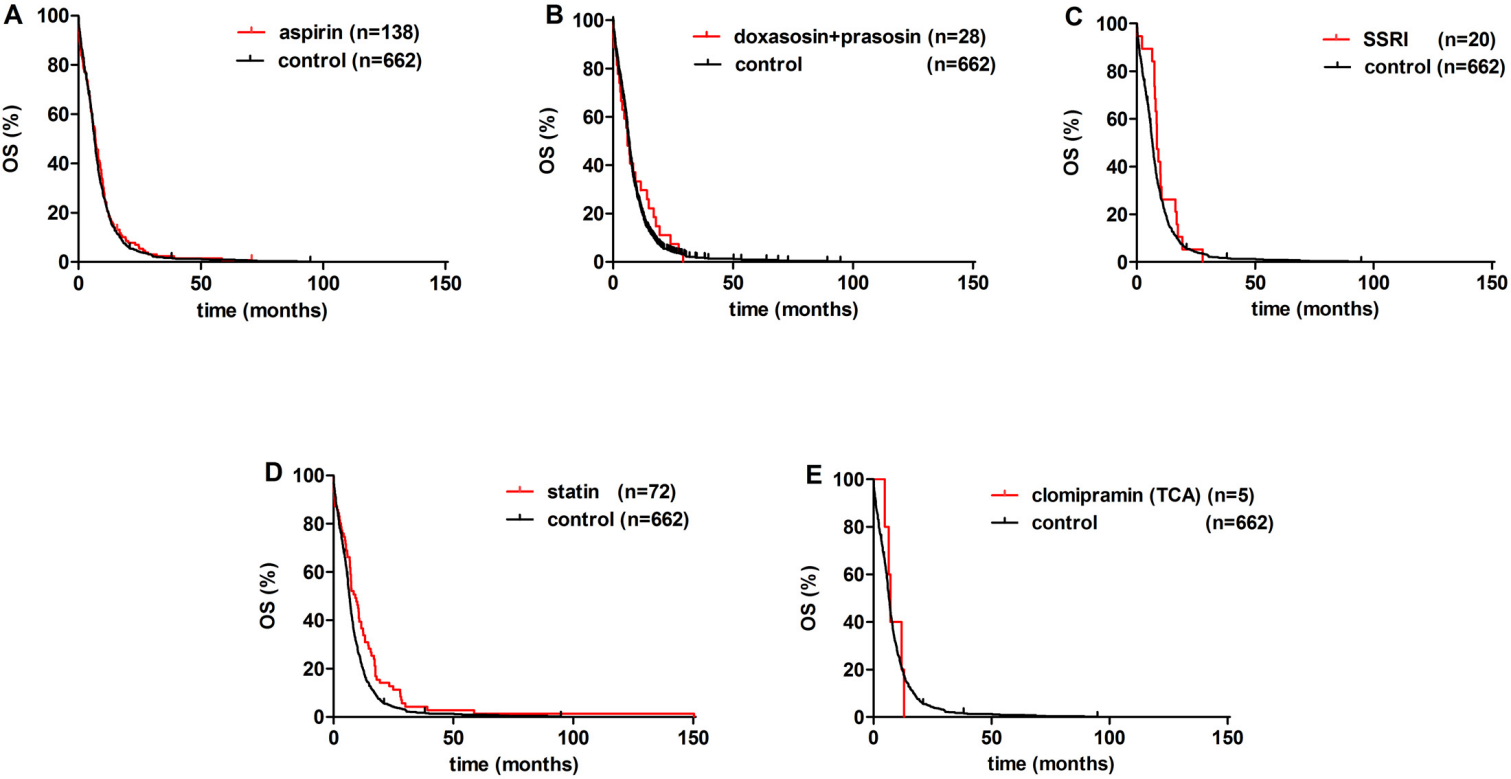
Kaplan-Meier curves for the OS of metastatic SCLC patients. Effect of FDA approved drugs. (A) Aspirin (vs. control; p = 0.225), (B) doxazosin and prazosin (vs. control; p<0.479), (C) selective serotonin reuptake inhibitors (SSRIs) (vs. control; p = 0.195), (D) statin treatment vs. control (p = 0.002) and, (E) clomipramine (a tricyclic antidepressant; TCA) vs. control had no effect on OS.

Univariate analysis with Bonferroni’s correction identified age, ECOG PS, statin treatment, and radiation therapy as significant prognostic factors (Figs 1 and 2, Tables 2 and 3). Age< 70 yrs (vs. ≥70 yrs) conferred a significantly improved OS (p<0.001; Fig 1A). Patients with ECOG PS 0–1 had significantly better OS than those presenting with ECOG PS >1 (p<0.001; Fig 1B). Among medications of various non-malignant disorders, a statistically significant increase in OS was observed in statin-prescribed patients when compared to those not prescribed any of the aforementioned medications (median OS, 8.4 vs. 6.1 months; respectively; p = 0.002; Fig 2D, Tables 2 and 3).

**Table 2:** Univariate survival analysis of studied drugs in metastatic SCLC. (n = 876).

	Univariate		
Prognostic factor	HR	95% CI	p-value
**aspirin**	0.9443	0.7854–1.135	0.5423
**SSRIs**	0.7765	0.5198–1.160	0.2165
**ADRA1**	0.9032	0.6244–1.306	0.5886
**statins**	1.477	1.156–1.890	0.002
**TCA**	0.9572	0.4036–2.271	0.921

*SSRIs*: selective serotonin reuptake inhibitors; *ADRA1*: α1-adrenergic receptor antagonists (doxazosin and prazosin), *TCA*: tricyclic antidepressant (clomipramine).

Two-sided log-rank tests were used for univariate survival analyses

**Table 3:** Clinical variables and survival of patients with metastatic SCLC in the Cox proportional hazards model.

	Univariate			Multivariate		
Prognostic factor	HR	95% CI	p-value	HR	95% CI	p-value
**Age (<70 yrs vs. ≥70 yrs)**	1.590	1.341–1.886	<0.001	1.358	1.119–1.647	0.002
**ECOG PS (0–1 v >1)**	1.675	1.452–1.931	<0.001	1.652	1.412–1.933	<0.001
**Statins**	1.477	1.156–1.890	0.002	1.094	0.851–1.408	0.483
**Radiation therapy**	2.160	1.876–2.500	<0.001	2.151	1.828–2.525	<0.001

***HR***: hazard ratio; ***CI***: confidence interval; ***ECOG PS***: Eastern Cooperative Oncology Group performance status.

Next, the clinicopathological characteristics were compared in the statin versus the control group (S1 Table). Patients with a statin prescription had significantly better performance status (ECOG PS 0–1, 76% vs. 63%, P = 0.03) and received significantly more first line platinum-etoposide therapy, PCI, and WBRT compared to the control group (78% vs. 61%; 13% vs. 2%; 33% vs. 19%; p values were below 0.001 in all cases). Prescriptions for statins were significantly associated with those patients receiving 2^nd^ line chemotherapy compared to the control group (53% vs. 39%, P = 0.025, Chi-square test, S2 Table). In contrast, treatment with other classes of drugs was not more frequent in patients receiving 2^nd^ line chemotherapy.

Furthermore, significantly increased median OS was observed in patients received RT when compared to those did not receive RT (10.0 vs. 4.8 months; respectively; p<0.001; Fig 1D). In contrast, the administration of SSRIs, aspirin, and ADRA1s did not result in a statistically significant OS benefit (median OS values were 8.5, 6.8, and 6.0, respectively (vs. 6.1 months in controls; Fig 2). The median OS was 7.2 months in the clomipramine group. Additionally, there was no significant difference in the OS by gender (Fig 1C). The multivariate Cox model showed that besides age and ECOG PS, RT was an independent survival predictor (HR, 2.151; 95% CI, 1.828–2.525; p<0.001; Table 3).

Next, the clinicopathological characteristics were compared in the RT vs. the non-RT group (Table 1). Patients received RT had significantly better performance status (ECOG PS 0–1, 73% vs. 63%; p = 0.003; Table 1), had younger age (59 vs. 62 yrs; p<0.001; Table 1), and received significantly more platinum-etoposide therapy (75% vs. 52%; respectively; p<0.001; Table 1) compared to all other patients.

## Discussion

In the last three decades, no new class of systemic therapy has entered clinical practice for advanced SCLC. Therefore, our study represents a strategy designed to evaluate a recent systematic drug repositioning bioinformatics study and different FDA approved medications with preclinical anti-SCLC activity in a clinical setting [6, 9, 10, 13]. This is one of the largest analyses of a well-defined metastatic SCLC cohort with a long-term follow-up. We analyzed whether the prescription of various medications was associated with survival benefit in a Hungarian cohort of patients, from a region with a high prevalence of lung cancer [2].

In this study, among reported standard prognostic parameters, only age <70 yrs and ECOG PS 0–1 proved to be an independent prognostic factor associated with longer OS similar as reported by others [15]. Female gender was not an independent survival predictor [16]. The increase in median OS remained significant in the multivariate analysis among patients received RT (besides age and ECOG PS). However, these patients had significantly better performance status (ECOG PS 0–1, 73% vs. 63%; p = 0.003; Table 1), were on average younger (59 vs. 62 yrs; p<0.001; Table 1), and received significantly more platinum-etoposide therapy compared to those not on RT (75% vs. 52%; respectively; p<0.001; Table 1). To date, increased OS for chemotherapy in SCLC was observed only in elderly patients [17]. The most recent NCCN guideline version I. 2015 reports no evidence of superiority among first-line chemotherapeutic agents. Our findings are in line with others that show that RT in SCLC is associated with survival benefit [18, 19]. The median OS of patients in the control group for medications listed in the methods section was 6.1 months, lower than OS from previously published data (7.1–9.4 months). This discrepancy is possibly due to the fact, that we included only metastatic patients (TanyNanyM1) in our study.”In the univariate analysis, a statistically significant increase in OS was observed in statin-prescribed patients when compared to the control group (median OS, 8.4 vs. 6.1 months; respectively; p = 0.002; Fig 1C). Of note, this difference did not remain significant in the multivariate analysis when RT was added as a confounding variable into Cox model. However, in a phase 2 study of 61 SCLC patients that received simvastatin in combination with irinotecan and cisplatin, there was improved survival in heavy smokers [16]. Furthermore, preclinical data in non-small cell lung cancer (NSCLC) reported that mTOR-dependent, statin-induced inhibition of Akt phosphorylation and nuclear translocation sensitizes cells to etoposide and other cytostatic drugs which supports our clinical findings [20]. This is in line with a very recent survey from Ireland, reporting that lung cancer patients prescribed simvastatin had reduced rates of cancer-specific mortality [12]. In contrast, a recent trial found no protective effect of pravastatin and chemotherapy compared with chemotherapy alone in SCLC patients [21]. Other previous reports were unable to demonstrate a statistically significant survival benefit [16, 22].

In the group of patients prescribed aspirin, the median OS was not significantly increased (6.8 vs. 6.1 months in control; Fig 2A). This finding is in line with a recent drug repositioning bioinformatical study that did not identify aspirins as candidate drugs with predicted efficacy against SCLC [6] or an earlier study that found no effect of aspirin on tumor progression [23]. A study from Poland in unilateral SCLC found a 1.5 fold greater probability of survival in patients receiving various antiplatelet drugs, including aspirin [24]. This discrepancy could be explained by the lower stage of disease analyzed compared to our population.

Of note, based on our results and number of patients in subgroups, we were not able to prove any significant survival benefit with prescribed medications acting on the GPCRs considered to be most relevant in SCLC by in vitro, in vivo, and in silico approaches [6]. Bearing in mind some limitations of the study, neither selective agents nor medications acting on multiple targets with the highest expectations in efficacy appeared to have increased OS. SSRIs did not show a significant increase of median OS (vs. control; 8.5 vs. 6.1 months; respectively; Fig 2C). We identified only 5 patients in the multitarget GPCR and monoamine transporter blocker clomipramine group (vs. control; median OS, 7.2 vs. 6.1 months; respectively; Fig 2E). Based on this limitation, we were not able to perform a statistical analysis, and therefore, to draw a firm conclusion. In a recent phase IIa clinical trial of second-line desipramine, there was rapid tumor progression and no clinical benefit for five patients with high grade neuroendocrine tumors [25, 26]. The investigators hypothesized that the different type of TCAs administered may have led to discordant outcome with preclinical findings (imipramine, clomipramine). The neurocognitive side effects led to intermittent and early discontinuation of the treatments and closure of the trial.

Our results show that ADRA1 antagonists did not appear to improve OS (median OS, 6.0 months (vs. 6.1 months in controls)). Our findings are in contrast to in silico data that demonstrated anti-SCLC activity [6]. This discrepancy is possibly caused by the unique biology, heterogeneity, and broad spectrum of neuroendocrine patterns of SCLC. Additional reasons to include: differences in the cancer microenvironment, and the administration and metabolism of the drug in vivo compared to the human body.

Due to its retrospective nature our study has several limitations. We were not able to verify medication compliance and duration of use. In addition, statins were prescribed more frequently during the latter years of this study period coinciding with the advance of the supportive oncology care, which may have contributed to the increased survival of those patients. Furthermore, it remains unclear whether a statin itself confers a more benign behavior of SCLC, decreases cardiovascular morbidity and mortality, or access to minimally invasive cardiology procedures which overlaps during the time period with more frequent statin prescribing patterns leads to improved survival. Furthermore, we did not have data on clinical depression, and thus, we were not able to use a more appropriate control group (e.g. patients with untreated major depression) for patients on antidepressants. This would have been of particular interest since it is well known that depression may negatively influence cancer patient survival [27]. Accordingly, it is conceivable that in our sample the possible survival benefit associated with antidepressant (TCA or SSRI) treatment was offset by depression itself. Although, compared to the control group OS values were also longer for the SSRIs and the TCA subgroups, the sample sizes were especially small in these two subgroups, which should also be considered as a possible limitation. Finally, compared to the control group a significantly higher proportion of subjects within the statin group received RT (a treatment modality results in prolonged survival in SCLC) which may decrease the validity of our conclusion on the positive effect of statins on survival [18][19].

Improving overall survival in SCLC is extremely difficult and has plagued drug development for this disease. It further explains the reason that no new class of systemic therapy has entered clinical practice over decades. Therefore, and more importantly, our study addresses an important issue in a unique way. After accounting for several limitations, among them the retrospective nature of our study, statins appear to provide a statistically significant survival benefit in the univariate analysis in metastatic SCLC. However, in the multivariate setting among therapies only RT appears to be an independent prognosticator for increased OS. Of course, studies with prospective designs and appropriately matched control subjects are needed to confirm our results on the beneficial effect of statin administration on the course of SCLC. Other classes of medications analyzed in this study did not validate the results of preclinical drug repositioning studies previously reported, suggesting that the results of drug repositioning studies using only preclinical data or small numbers of patients should be treated with caution before application in the clinic.

## Supporting Information

S1 Table Major clinicopathological characteristics of patients received FDA- approved drugs of various non-malignant disorders reported to have potential anti-SCLC activity. Data shown in parentheses are column percentages. *ECOG PS*: Eastern Cooperative Oncology Group performance status; *OS*: overall survival; *TCA*: tricyclic antidepressant (clomipramine), *SSRIs*: selective serotonin reuptake inhibitors; *CEV*: cyclophosphamide, epirubicin, vincristine; *PCI*: prophylactic cranial irradiation; *WBRT*: whole brain radiation therapy; *ADRA1*: α1-adrenergic receptor antagonists (doxazosin and prazosin).
(PDF)

S2 Table Distribution of prescribed study drugs according to the different lines of chemotherapy*. *SSRIs*: selective serotonin reuptake inhibitors; *ADRA1*: α1-adrenergic receptor antagonists (doxazosin and prazosin), *TCA*: tricyclic antidepressant (clomipramine), *CHT*: chemotherapy. Two-sided log-rank tests were used for univariate survival analyses. *there was no data in the case of 12 patients on chemotherapy administration and 61 patients received best supportive care.
(PDF)
