# Supplementary material for: From Bench to Bedside: Attempt to Evaluate Repositioning of Drugs in the Treatment of Metastatic Small Cell Lung Cancer (SCLC)
Source: PLoS One. 2016 Jan 6;11(1):e0144797. doi: 10.1371/journal.pone.0144797 (PMC4703211; doi:10.1371/journal.pone.0144797)
Supplement: S1 Table — Data shown in parentheses are column percentages. ECOG PS: Eastern Cooperative Oncology Group performance status; OS: overall survival; TCA: tricyclic antidepressant (clomipramine), SSRIs: selective serotonin reuptake inhibitors; CEV: cyclophosphamide, epirubicin, vincristine; PCI: prophylactic cranial irradiation; WBRT: whole brain radiation therapy; ADRA1: α1-adrenergic receptor antagonists (doxazosin and prazosin). (PDF) [file pone.0144797.s001.pdf]

		aspirin	SSRIs	ADRA1	statins	TCA	control
**Total**		138	20	28	72	5	662
**Age (mean±SD)**		65±8.2	59±9.8	65±8.2	63±8.7	56±5.9	61±9
**Gender**	**Male**	80(58%)	6(30%)	12(43%)	36 (50%)	2(40%)	395(60%)
	**Female**	58(42%)	14(70%)	16(57%)	36 (50%)	3(60%)	267(40%)
**ECOG performance status**	**0-1**	98(72%)	15(75%)	17(61%)	54 (76%)	4(80%)	401(63%)
	**>1**	38(28%)	5(25%)	12(39%)	17 (24%)	1(20%)	234(37%)
	**Unknown data**	2	0	0	1	0	27
**Chemotherapy**	**Platinum-etoposide**	101(77%)	10(56%)	19(76%)	56 (78%)	3(60%)	368(61%)
	**CEV**	31(23%)	8(44%)	6(24%)	10 (14%)	2(40%)	235(39%)
	**Unknown data/ best supportive care**	6	2	3	6 (8%)	0	59
**Radiation therapy**	**PCI**	10(7%)	0(0%)	1(10%)	9 (13%)	0(0%)	14(2%)
	**WBRT**	25(18%)	9(45%)	5(50%)	24 (33%)	1(20%)	126(19%)
	**Thoracic RT**	18(13%)	8(40%)	4(40%)	12 (17%)	2(40%)	122(18%)
**Median OS (95%CI) months**		6.8(5.5-8.2)	8.5 (8.3-8.8)	6.0 (3.2.-8.8)	8.4 (5.2-11.5)	7.2 (5.8-8.5)	6.1 (5.7-6.5)
