# Supplementary material for: From Bench to Bedside: Attempt to Evaluate Repositioning of Drugs in the Treatment of Metastatic Small Cell Lung Cancer (SCLC)
Source: PLoS One. 2016 Jan 6;11(1):e0144797. doi: 10.1371/journal.pone.0144797 (PMC4703211; doi:10.1371/journal.pone.0144797)
Supplement: S2 Table — SSRIs: selective serotonin reuptake inhibitors; ADRA1: α1-adrenergic receptor antagonists (doxazosin and prazosin), TCA: tricyclic antidepressant (clomipramine), CHT: chemotherapy. Two-sided log-rank tests were used for univariate survival analyses. *there was no data in the case of 12 patients on chemotherapy administration and 61 patients received best supportive care. (PDF) [file pone.0144797.s002.pdf]

	1st line CHT	2nd line CHT	p value
**total number of patients**	139 (57%)	107 (43%)	0.206
**aspirin**	79 (60%)	53 (40%)	0.774
**SSRIs**	7 (39%)	11 (61%)	0.084
**ADRA1**	19 (76%)	6 (24%)	0.148
**statins**	31 (47%)	35 (53%)	**0.025**
**TCA**	3 (60%)	2 (40%)	-
**control only CHT without repurposed drugs**	369 (61%)	234 (39%)	-
